# Bone Formation in Unilateral Cleft Lip and Palate Patients after Early Secondary Gingivoalveoloplasty and Bone Graft: Long-Term Study

**DOI:** 10.1097/PRS.0000000000011684

**Published:** 2024-08-20

**Authors:** Luca Autelitano, Daniele Hamaui, Valeria M. A. Battista, Federico Biglioli, Maria C. Meazzini

**Affiliations:** Milan, Italy; From the Cleft Lip and Palate Regional Center, Operation Smile, San Paolo University Hospital, ASST Santi Paolo e Carlo Milano.

## Abstract

**Background:**

The aim of this study was to conduct a comparative analysis, using computed tomographic scans, of ossification patterns in unilateral cleft lip and palate patients who underwent early secondary gingivoalveoloplasty (esGAP) versus those who underwent traditional alveolar bone grafting harvested from the iliac crest (IC).

**Methods:**

Computed tomographic scans of 22 consecutively treated patients with esGAP were compared with those of 21 patients treated with bone grafting from the IC. Inclusion criteria were nonsyndromic unilateral cleft lip and palate patients in permanent dentition. Two parameters were considered: the alveolar thickness, measured at 3 levels, and the nasoalveolar height. All measurements were normalized and ratios of the affected versus nonaffected sides were provided as in addition to the statistical comparison between the 2 groups’ ossification outcomes.

**Results:**

In the esGAP sample, nasoalveolar height was categorized as ideal and good in 86.36% and in 13.64% of the cases, and no mediocre or insufficient ossification was detected; whereas in the bone grafting sample, 38.10% had ideal and good ossification, 14.29% had mediocre ossification, and 9.52% had insufficient ossification. As regards the alveolar thickness, when we consider the average of 3 levels, the esGAP sample was ideal and good in 57.57% and in 30.30% of the cases; for the IC sample, the alveolar thickness was ideal and good 41.27% and in 25.40% of the cases, respectively. The analysis detected a statistically significant difference in the ossification outcomes in the 2 samples.

**Conclusion:**

The esGAP yields superior ossification grades in comparison to IC bone grafting.

**Clinical Relevance Statement:**

In unilateral cleft lip and palate patients, esGAP seems to allow for a higher grade of ossification compared to IC bone grafting.

**CLINICAL QUESTION/LEVEL OF EVIDENCE:**

Therapeutic, III.

Numerous techniques have been devised to ensure adequate bone quantity and quality in the cleft area for tooth eruption, primarily targeting maxillary canine or lateral incisor. However, the choice of the timing and of the specific surgical procedures remain subjects of debate.

Several approaches for the primary closure of the alveolar cleft have been proposed. In 1980, Millard advocated for primary gingivoperiosteoplasty (GPP) at 3 months of age,^1^ coinciding with lip repair. Delaire suggested early secondary gingivoalveoloplasty (esGAP), delaying surgery until 18 months of age, during hard palate repair.^2^ Secondary bone grafting is widely regarded as the standard procedure for alveolar repair in many surgical protocols.^3^

In our cleft lip and palate (CLP) center, our surgical protocol comprises the following stages: lip, nose, and soft palate repair typically occur at 4 to 6 months of age and the second step, between 18 to 24 months, involves simultaneous repair of the hard palate along with an esGAP performed on the untouched alveolar tissue, deviating from Delaire’s approach, and without any presurgical orthopedic treatment. This is possible because of the molding effect on the maxillary ridges of the repaired lip.^4^ The vestibular flap is prepared according to the procedure described by Boyne and Sands^3^ for secondary bone grafting. An undermining of the vestibular and palatal flaps subperiostally, and of the mucoperiosteal flaps of the nasal side, is performed, allowing the nasal layer suture to form the posterior part of the hard palate. A hermetic suture of the nasal layer is fundamental for a good ossification in the newly created pyramidal space extended to the base of the apertura piriformis. Ossification studies have demonstrated 95% of success after esGAP.^5^

In patients treated primarily in other centers, who have a residual alveolar cleft, we performed bone grafting either just before the eruption of the lateral incisor,^6^ or of the canine,^7^ when their roots have reached two-thirds of development.^8^ Because it is well known that the eruption of the tooth is one of the most important factors influencing the outcome of bone grafting,^9–11^ this timing offers an ideal sagittal and vertical reconstruction. Iliac crest bone is one of the most popular donor sites, given the possibility of harvesting a large amount of cancellous bone.^12^ Many other sources of bone have been used such as the calvaria, tibia,^13^ the ribs,^14^ the vomerine bone,^15^ and the mandibular symphisis,^16^ with the latter having the advantage of a single operative field, no visible scar, and less postoperative pain.^17^ The main goal of this study was to compare three-dimensionally the alveolar bone formation in a group of patients who underwent IC secondary bone grafting with the one in a group of patients treated with esGAP.

## PATIENTS AND METHODS

### Patient Cohort

The study was conducted following the ethical principles outlined in the World Medical Association Declaration of Helsinki. The analysis was undertaken with the understanding and written consent of each individual and relative parents and according to the above-mentioned principles.

A single-center retrospective study was conducted on nonsyndromic patients affected by complete unilateral cleft and lip palate (UCLP) with an untreated alveolar bone. To standardize the great heterogeneity of these patients, exclusion criteria were all bilateral CLPs, incomplete unilateral CLPs, CLPs in syndromic children, and UCLP patients who had undergone a previous alveolar surgery. Moreover, low-quality computed tomographic (CT) scans were excluded from our study.

There was no patient selection based on clinical severity indicators such as cleft width or lateral incisor agenesis. The study included 43 patients divided into 2 groups (Table 1). The first group consisted of 22 nonsyndromic UCLP patients who underwent esGAP. They were treated consecutively according to the 2-stage protocol of our center, by the senior surgeon (L.A.). The second group consisted of 21 nonsyndromic unilateral cleft lip and palate patients who had previously received primary treatment at other medical centers and were referred to us for secondary bone grafting. These patients underwent alveolar bone grafting from the iliac crest performed by the same surgeon (L.A.) at our center.

**Table 1.** Demographic and Unilateral Cleft Lip and Palate Data

Characteristic	esGAP (%)	IC (%)	*P*
No.	22	21	
Sex			
Male	17 (77.27)	16 (76.19)	
Female	5 (22.73)	5 (23.81)	
Age at CT			
Median	15.56	11.83	
IQR	13.20–16.37	9.97–12.31	0.000^a^^,^^b^
Age at surgery			
Median	2.62	10.13	
IQR	2.00–3.11	8.79–10.50	0.000^a^^,^^b^
Laterality			0.663^c^
Right	14 (63.64)	12 (57.14)	
Left	8 (36.36)	9 (42.86)	

IQR, interquartile range.

a Statistically significant.

b Independent-samples Kruskal Wallis test, pairwise comparisons.

c χ^2^ test.

### Data Collection

Alveolar cleft ossification was quantitatively and qualitatively evaluated on CT images. Quantitative analyses provided both absolute normalized measures and ratios of the affected side versus the nonaffected side. The ratios were classified according to the modified Bergland scoring system.^18^ We compared cleft and noncleft sides using the method described by Meazzini et al.^5^: a midline running through the center of the cervical vertebra and the airways was drawn, and the thinnest area on the cleft side and its mirror section on the noncleft side were selected.

**Fig. 1.**
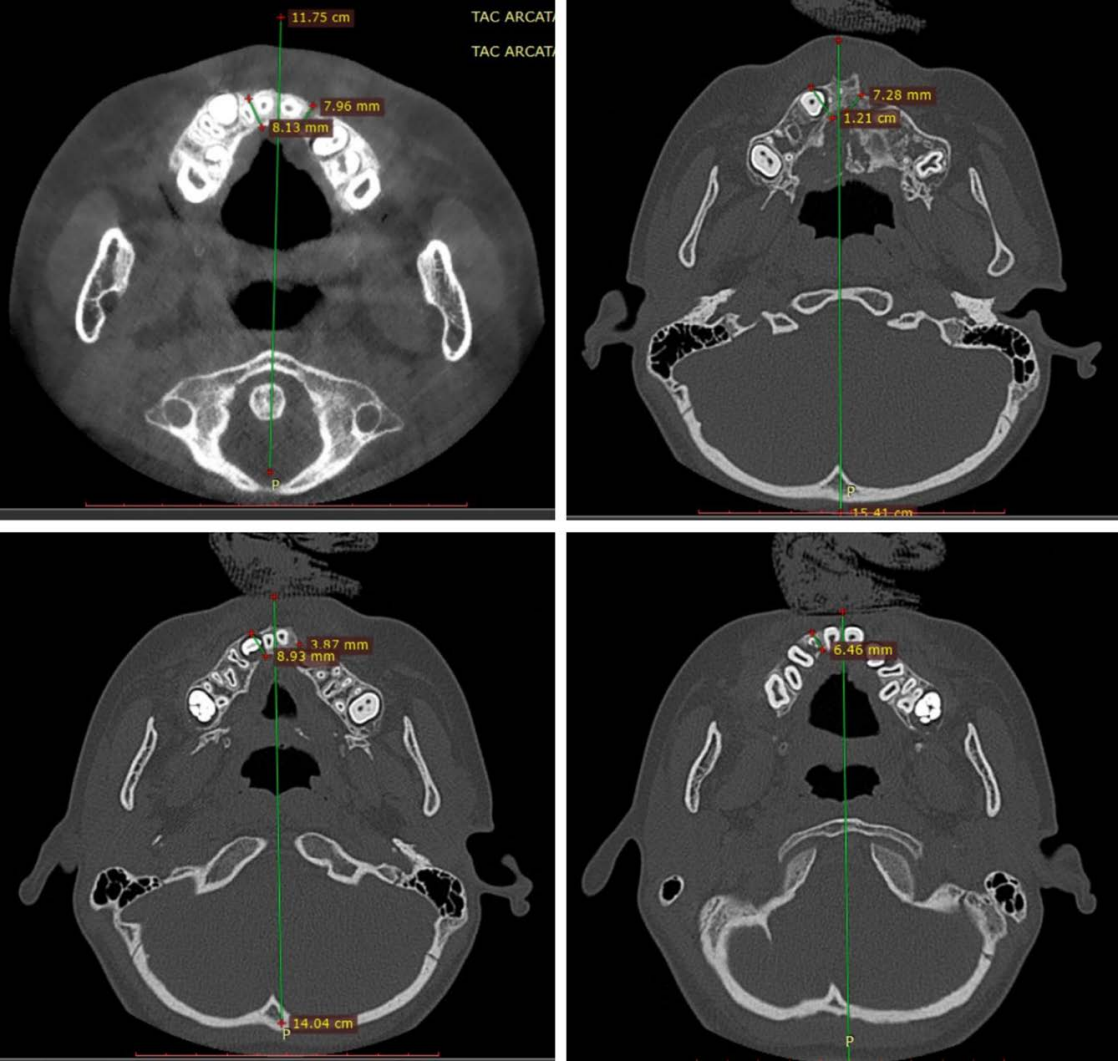
Axial CT scans show different degrees of alveolar ossification depending on the calculated ratio of the cleft versus noncleft side. (*Above*, *left*) Ideal ossification: from 100% to 75%. (*Above*, *right*) Good ossification: from 75% to 50%. (*Below*, *left*) Mediocre ossification: from 50% to 25%. (*Below*, *right*) Insufficient ossification: from 25% to 0%.

To provide a reliable evaluation, 3 different levels from palatal to vestibular cortical bone were considered for alveolar thickness (AT), measured using both dental and axial scans: a high nasal level, an imaginary plane crossing the apex of the permanent central incisor; a low gingival level, passing though the enamel-dentinal junction; and an intermediate level, equidistant from these two planes. Depending on the calculated ratio of the cleft versus noncleft side,^19^ the obtained data were classified as ideal from 100% to 75%, good from 75% to 50%, mediocre from 50% to 25%, and insufficient from 25% to 0% (Fig. 1).

**Fig. 2.**
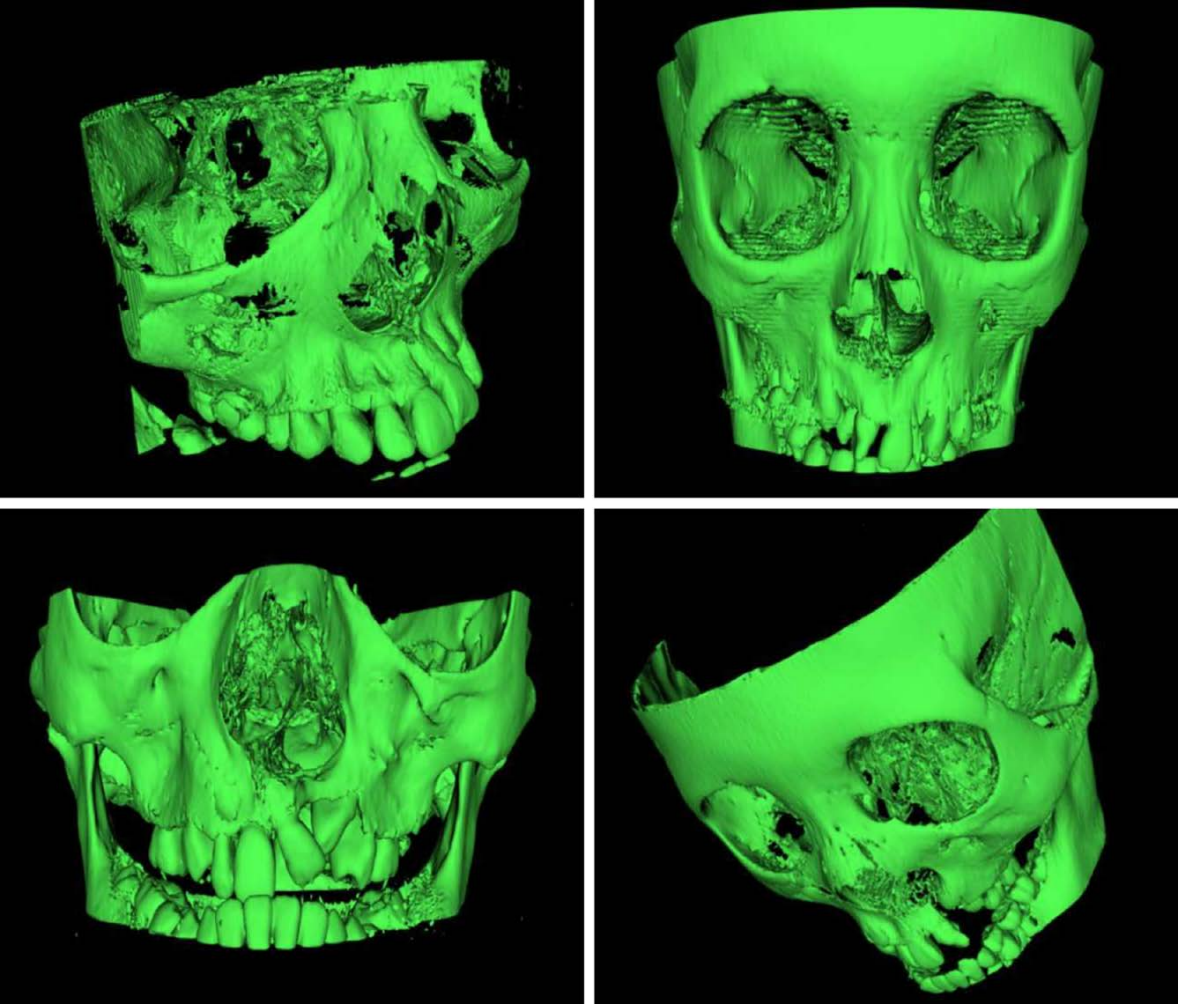
Three-dimensional model reconstructions of the 4 ossification classes previously shown. (*Above*, *left*) Ideal ossification: from 100% to 75%. (*Above*, *right*) Good ossification: from 75% to 50%. (*Below*, *left*) Mediocre ossification: from 50% to 25%. (*Below*, *right*) Insufficient ossification: from 25% to 0%.

Nasoalveolar height (NAH) was measured using both dental scans and three-dimensional images, indicating the vertical distance between the most alveolar occlusal point and the nasal floor. As regards the NAH parameter, the measurements were ranked using the same 4 classes of outcomes depending on the calculated ratios of the cleft versus noncleft side (Fig. 2). All measurements were repeated twice and by 2 different researchers.

### Statistical Analysis

Statistical analysis was performed using STATA (STATA/SE 15.1). To check the statistical significance of the difference in alveolar ossification, between the cleft side and the healthy side, within the 2 groups, 2 tests were performed. First, a paired *t* test was computed using absolute values of the measurements of both AT and NAH. Second, a Spearman rho (ρ) test was used to evaluate the strength of the correlation between the NAH and the 3 cuts of the AT computing the previously mentioned ratios that we classified into 4 classes of success (ideal, good, mediocre, or insufficient).

Then, we performed a between sample comparison. To test any significant difference between the esGAP and the IC bone grafting samples, we used a Mann-Whitney *U* test, which is a more robust test when the data do not follow a normal distribution.

Furthermore, a Pearson chi-square test was run to test the difference in the median of the distributions of the ossification in the esGAP and IC bone grafting samples. Finally, after rearranging the results in dichotomous classes of outcomes (success if ossification reached an ossification of greater than or equal to 75% and failure when less than 75%), Goodman-Kruskal gamma and Fisher exact tests were performed. These tests, that are particularly effective in samples with a limited number of observations, were used to investigate whether any nonrandom association between the two dichotomous categorical variables (success or failure) could be assessed. Finally, an ordered logistic regression analysis was conducted to ascertain whether there exists a significant association between the degree of ossification in the esGAP and IC bone grafting groups and the timing of surgical intervention.

## RESULTS

### Demographic Data

The entire cohort consisted of 33 boys (76.74%) and 10 girls (23.26%). There was no statistically significant difference in gender breakdown between groups (Table 1). As regards the esGAP sample, 22.73% were girls and 77.27% were boys; 36.36% of the clefts were on the left side and 63.64% were on the right side. The median age at the time of esGAP was 2.62 years (interquartile range [IQR], 2.00 to 3.11 years). The assessment was conducted on completion of full permanent dentition, when CT scans were needed for other orthodontic/orthopedic reasons or for planning of rhinoplasty. The computed tomographic scans were limited to the maxilla to minimize radiation exposure. The median age at the time of the assessment was 15.56 years (IQR, 13.20 to 16.37 years). In this sample, the median time elapsed between esGAP and CT scan assessment was 12.97 (IQR, 10.09 to 14.00 years).

In the IC cohort, 23.81% were girls and 76.19% boys; 42.86% of the clefts were on the left side and 57.14% were on the right side, and no statistical difference in laterality distribution was detected when compared with the esGAP group. The median age at the time of alveolar bone grafting was 10.13 years (IQR, 8.79 to 10.50 years). The median age at the time of assessment was 11.83 years (IQR, 9.97 to 12.31 years). The median time elapsed between bone grafting and the CT assessment was 1.33 years (IQR, 1.12 to 1.70 years).

### Measurements

Tables 2 and 3 summarize all measurements, presented as percentage values comparing the affected side to the nonaffected side, for both the esGAP and the IC bone grafting samples. The NAH, in the esGAP sample, was classified as ideal in 86.36% of the cases (19 of 22 patients), and good in 13.64% of the cases. No mediocre or insufficient ossification was detected. In the IC bone grafting sample, 38.10% of the patients (8 of 21 patients) reported an ideal ossification and an equal fraction a good ossification, whereas 14.29% and 9.52% (3 and 2 of 21 patients) were classified as mediocre and insufficient, respectively.

**Table 2.** All Measurements Comparing the Affected Side with the Nonaffected Side of NAH and AT in the esGAP Sample (*n* = 22)

esGAP	AT^a^				NAH (%)^b^
	High (%)	Intermediate (%)	Low (%)	Average (%)	
Ideal (75%–100%)	72.73	63.63	36.36	57.57	86.36
Good (50%–75%)	22.72	31.82	36.36	30.30	13.64
Mediocre (25%–50%)	0	4.55	4.55	3.03	0
Insufficient (0%–25%)	4.55	0	22.73	9.08	0

a (Cleft alveolar thickness/noncleft alveolar thickness) × 100.

b (Cleft alveolar height/noncleft alveolar height) × 100.

**Table 3.** All Measurements Comparing the Affected Side with the Nonaffected Side of Nasoalveolar Height and Alveolar Thickness in the IC Bone Grafting Sample (*n* = 21)

IC Bone Graft	AT^a^				NAH (%)^b^
	High (%)	Intermediate (%)	Low (%)	Average (%)	
Ideal (75%–100%)	47.62	61.90	14.29	41.27	38.10
Good (50%–75%)	38.10	19.05	19.05	25.40	38.10
Mediocre (25%–50%)	9.52	9.52	14.29	11.11	14.29
Insufficient (0%–25%)	4.76	9.52	52.36	22.21	9.52

a (Cleft alveolar thickness/noncleft alveolar thickness) × 100.

b (Cleft alveolar height/noncleft alveolar height) × 100.

The average value for the 3 levels of alveolar thickness, in the esGAP sample, was classified as ideal in 57.57%, good in 30.30%, mediocre only in 3.03%, and insufficient in 9.08% of the cases. In the IC bone grafting sample, AT was ideal in 41.27%, good in 25.40%, mediocre in 11.11%, and insufficient in 22.20% of the cases.

### In-Group Analysis

In both samples, a paired *t* test unequivocally demonstrated the statistical significance, in absolute terms, of the discrepancy between the cleft side and the healthy side across each parameter (*P* < 0.000). Table 4 indicates that the Spearman rho test revealed no statistically significant association between NAH and AT at the 3 levels for esGAP. Conversely, in Table 5, the Spearman rho test for IC highlighted a lack of statistically significant association only between NAH and the low AT level, but it did reveal a statistically significant association between NAH and the high and intermediate AT levels (*P* < 0.05).

**Table 4.** Spearman Rank Correlation Coefficient Computed to Investigate Any Statistical Relationship between NAH and AT at 3 Levels within the esGAP Group^a^

	esGAP		
	ρ	95% CI	*P*
NAH vs. AT high	−0.241	−0.452 to −0.117	0.278
NAH vs. AT intermediate	−0.297	−0.516 to −0.149	0.179
NAH vs. AT low	0.055	−0.347 to 0.486	0.807

a The Spearman rho (ρ) test used percentage measurements of ossification for each parameter.

**Table 5.** Spearman Rank Correlation Coefficient Computed to Investigate Any Statistical Relationship between NAH and AT at 3 Levels within the IC Bone Grafting Group^a^

	IC Bone Graft		
	ρ	95% CI	*P*
NAH vs. AT high	0.537	0.119–0.804	0.012
NAH vs. AT intermediate	0.555	0.154–0.831	0.009
NAH vs. AT low	0.194	−0.298 to 0.626	0.399

a The Spearman rho (ρ) test used percentage measurements of ossification for each parameter.

### Between-Group Analysis

The comparison between the 2 surgical procedures was performed with a Mann-Whitney *U* test for the ratios of each parameter, showing a statistically significant higher grade of ossification in the esGAP sample for the low AT and the NAH (NAH, IC bone grafting versus esGAP *u* −4.082 [*P* = 0.000]; high AT, IC bone grafting versus esGAP *u* −0.875 [*P* = 0.381]; intermediate AT, IC bone grafting versus esGAP *u* −0.523 [*P* = 0.601]; low AT, IC bone grafting versus esGAP *u* −2.333 [*P* = 0.019]).

A Pearson chi-square test showed that the median of the distributions of the 2 groups was significantly higher in esGAP patients in terms of both NAH and low AT level (NAH median of esGAP versus IC bone grafting chi-square 10.2897 [*P* = 0.001]; low AT median of esGAP versus IC bone grafting chi-square 6.746 [*P* = 0.009]). It was not statistically significantly different at intermediate and high AT levels (high AT median of esGAP versus IC bone grafting chi-square 0.587 [*P* = 0.443]; intermediate AT median of esGAP versus IC bone grafting chi-square 0.206 [*P* = 0.650]).

Moreover, the Goodman-Kruskal gamma and Fisher exact tests indicated that the percentage of values at the low, intermediate, and high levels of AT exhibited no statistically significant correlation, as demonstrated in Table 6. Finally, the timing of surgical intervention was not significantly related to the classification of alveolar ossification, NAH, and AT, at all levels (Tables 7 and 8).

**Table 6.** Goodman Kruskal and Fisher Exact Test for Each of the Parameters^a^

	AT High		AT Intermediate		AT Low		NAH	
	Success	Failure	Success	Failure	Success	Failure	Success	Failure
esGAP (*n* = 22)	16	6	14	8	8	14	19	3
IC graft (*n* = 21)	10	11	13	8	3	18	8	13
Goodman Kruskal	0.491		0.037		0.548		0.822^b^^,^^c^	
*P* Fisher	0.124		1.000		0.162		0.002	

a The data were classified in dichotomous groups where success means an ossification ≥75% and failure <75%.

b Statistically significant.

c *P* < 0.001.

**Table 7.** Estimates of an Ordered Logistic Model of the Timing of Surgical Intervention, Expressed in Terms of the Average Adjusted Predictions for the esGAP Sample (*n* = 22)

esGAP	AT				NAH
	High	Intermediate	Low	Average	
Ideal	0.007	−0.001	0.013	0.014	0.010
*P*	0.416	0.895	0.233	0.353	0.067
Good	−0.001	0.001	−0.010	−0.009	−0.010
*P*	0.669	0.896	0.248	0.362	0.067
Mediocre	−0.000	0.000	—	−0.004	—
*P*	0.490	0.889		0.433	
Insufficient	−0.005	—	−0.002	—	—
*P*	0.422		0.421	0.3584	
Prob > χ^2^	0.4100	0.8951	0.2098		0.0951

**Table 8.** Estimates of an Ordered Logistic Model of the Timing of Surgical Intervention, Expressed in Terms of the Average Adjusted Predictions for the IC Bone Grafting Sample (*n* = 21)

IC Bone Graft	AT				NAH
	High	Intermediate	Low	Average	
Ideal	−0.002	−0.002	−0.005	−0.002	−0.010
*P*	0.566	0.622	0.361	0.566	0.068
Good	−0.001	0.000	0.003	−0.001	0.002
*P*	0.595	0.621	0.383	0.595	0.438
Mediocre	−0.000	0.000	0.001	−0.000	0.004
*P*	0.639	0.591	0.370	0.639	0.124
Insufficient	0.004	0.001	0.000	0.004	0.003
*P*	0.568	0.672	0.557	0.568	0.270
Prob > χ^2^	0.5682	0.6204	0.3617	0.3137	0.0714

Statistical data serve as valuable indicators of the obtained results and facilitate comparison. However, in this specific type of surgery, the clinical interpretation of the outcomes holds paramount importance: we observed a high clinical success rate in both samples. Notably, none of the patients in this small sample required secondary bone grafting before phase 2 orthodontics. Conversely, 2 patients in the bone grafting group necessitated tertiary bone grafting to ensure safe orthodontic teeth movement.

## DISCUSSION

The management of alveolar clefts remains a contentious aspect of CLP treatment. The Bergland scoring system has commonly been used in assessing alveolar ossification after secondary bone grafting through plain radiographs.^19–21^ This method is widely accepted globally because of its ease of reproducibility and low radiation dose. A prior study by Meazzini et al. assessed ossification using panoramic radiographs, already available for orthodontic purposes, in a sample of patients who underwent esGAP, revealing that 85% of the patients exhibited type I ossification.^17,22^ However, some studies have suggested that panoramic radiographs may overestimate ossification by up to 20%.^23^ This issue is associated with image distortion and structural overlap.^24^ To improve accuracy, many authors proposed the use of CT scans.^25–29^ Computed tomography furnishes precise details concerning bone architecture, density, and three-dimensional thickness. However, this technique entails exposing the patient to a high radiation dose. Cone beam computed tomography (CBCT) has been more recently proposed to mitigate radiation exposure.^30–33^

Nevertheless, CBCT is not universally regarded as the standard because of the lack of highly standardized methods for image acquisition and reconstruction.^34^ In our study, both CBCT and multislice CT scans were used. Nonetheless, in our protocol, only CBCT scans were designated as the preoperative radiologic examination because of their low radiation dose and provision of high-quality images.

Although there may be some variability among different CBCT systems in visualizing fine structures, their image quality is comparable or even superior to multislice CT scanning.^35,36^ Therefore, as recommended by existing literature, both radiologic techniques can be used, provided that they provide high-quality images.

The primary limitations of our study lie in the retrospective nature of the collected data, which may introduce a potential bias in our results, and in the relatively low number of patients, attributed to the implementation of stringent selection criteria. The objective was to assess and compare the ossification outcomes between 2 groups: patients who received bone grafting and those who underwent esGAP. Following primary GPP, literature indicates a requirement for secondary bone grafting in 30% to 90% of patients.^37,38^ In addition, numerous studies have identified a significant deficiency in maxillary growth, leading to a heightened demand for orthognathic surgery.^39,40^

In contrast, esGAP seems to exhibit a higher rate of ossification. Meazzini et al.^5^ reported a complete failure of bone bridging in only 1.7% of the esGAP sample, and 3.8% of patients presenting an ossification lower than 25% of the noncleft side. Our study revealed a high percentage of surgical success in terms of alveolar ossification in both the IC bone grafting and esGAP samples. In addition, esGAP seemed to demonstrate superior ossification compared with bone grafting. Interestingly, ossification of esGAP seems to be more predictable than primary GPP. This finding was consistently observed in alveolar thicknesses, as supported by both the Mann-Whitney *U* and Pearson chi-square tests, suggesting that ideal and good ossification are more associated with esGAP than with IC-grafted patients. Furthermore, our study underscores 2 important aspects. First, it seems to affirm the reliability of the three-dimensional measurement method used by Meazzini et al.^5^ Second, a significantly strong association between NAH and AT was observed, indicating that esGAP facilitates robust bone formation in both height and thickness. Consequently, the results suggest that esGAP proves to be an effective procedure for addressing bone defects in the alveolar region. Although IC secondary bone grafting remains the standard for alveolar cleft repair, consistently yielding favorable ossification outcomes, esGAP seems to offer a more comprehensive anatomical reconstruction. However, this technique requires a careful restoration of the physiologic nasal, palatal, and vestibular mucoperiosteal plane; avoiding tissue tension; and creating a pyramidal space for bone growth. These outcomes are only achieved by performing the GPP, after lip and soft palate closure have provided alveolar molding. The procedure avoids a third surgical step of bone grafting at the age of the lateral incisor or of the canine eruption in the majority of patients.

In both cohorts, the operations were performed by the same surgeon. The esGAP was performed in the 2-stage protocol of our center. IC bone grafting was reserved for patients who had not undergone primary alveolar repair and were referred from other centers.

Another aspect to consider is the impact of esGAP on maxillary growth, which has been previously investigated in 2 other studies. The first study compared 2 patient groups treated with the same surgical protocol by the same surgeon. However, in the earlier group, no early secondary gingivoalveoloplasty was performed; instead, a secondary bone grafting procedure was carried out at the age of 9 to 10 years.^41^ Although early secondary gingivoalveoloplasty significantly reduced the necessity for secondary bone grafting, it had a restraining effect on maxillary growth, resulting in a heightened requirement for Le Fort I osteotomies. Conversely, the second study compared the long-term outcomes between the Milan and Oslo samples.^42^ The anticipated need for orthognathic surgery seemed to be greater in the Milan sample (26% versus 13%). However, in the Milan group, only patients requiring an additional orthognathic procedure have to undergo a third surgical step, whereas the Oslo protocol mandates 3 surgical steps for all patients. Consequently, this suggests that despite the negative impact on growth, the overall advantage of our protocol in terms of the burden of care may still favor early secondary GPP.

Finally, another potential parameter for evaluation is the pattern of canine eruption and the subsequent potential for tooth retention. In a previous study, we observed that 15.50% of patients exhibited canine retention, a figure lower than the majority reported in the literature, and 4.40% of individuals in the entire sample necessitated surgical exposure.^43^

Approximately 50% of patients in the bone grafting sample underwent maxillary expansion to align the lesser and larger segments and facilitate improved surgical access. Conversely, in the esGAP sample, over 85% of patients required expansion.

## CONCLUSION

Early secondary gingivoalveoloplasty yielded superior ossification grades in both alveolar thickness and height compared with bone grafting.

## DISCLOSURE

The authors have no financial interest in any of the products or devices mentioned in this article. This study was not supported by any company, institute, or organization that has profit-obtaining purposes.
